# Persistent transverse arch hypoplasia is associated with systemic hypertension after coarctation of aorta repair

**DOI:** 10.1186/1532-429X-17-S1-O59

**Published:** 2015-02-03

**Authors:** Sophie Quennelle, Ashwin Prakash

**Affiliations:** 1 Cardiology, Boston Children's Hospital, Boston, MA, USA

## Background

A high prevalence of systemic hypertension after coarctation repair has been shown to be related to vascular dysfunction. Although mild transverse aortic arch (TAA) hypoplasia is common after coarctation repair, its role in the development of hypertension remains unclear. We examined the association between persistent TAA hypoplasia and hypertension late after coarctation repair

## Methods

A retrospective review of clinical, CMR and exercise data on patients after coarctation repair was performed to include patients who had CMR from 2005-2014. Patients with atypical coarctation, major heart defects, residual coarctation (right arm-leg blood pressure gradient > 20 mm Hg) or >mild semilunar or atrioventricular valve stenosis/regurgitation were excluded. Patients satisfying standard criteria for resting hypertension and those on anti-hypertensive medication were classified as being "hypertensive". In a subset of patients, exercise testing was used to evaluate the increase in right arm systolic blood pressure (SBP) and arm-leg SBP gradient during peak exercise. CMR images were analyzed to calculate the minimum TAA diameter z-score, the ratio of TAA and thoracic descending aorta (DAO) diameters, isthmus diameter z-score and, LV mass/BSA.

## Results

We analyzed data on 92 patients (median age 19.8 years; 60% male) who had initial treatment at a median age of 4.6 (0-57) years using surgery (n=63), stenting (n=16) or balloon angioplasty (n=13). 31/92 (33%) required a second procedure. On recent follow-up, 38/92 (41%) patients had resting hypertension (n=11) or were on antihypertensive medication (n=30). On CMR, median TAA z-score was -2.1 (-4.0 to 1.1) and median TAA/DAO diameter ratio was 0.92 (0.43-1.27). Being in the "hypertensive" group was associated with a smaller TAA (p=0.002, odds ratio 1.5 for 0.1 point decrease in TAA/DAO diameter ratio) and higher age (p=0.04, odds ratio 1.05 for 1 year increase in age) but not with isthmus diameter z-score, age or type of repair or need for a second procedure. In a subset of patients with an available stress-test (n=61), a lower TAA/DAO diameter ratio was associated with a larger increase in right arm SBP during peak exercise (p=0.006, r^2^=0.12) but not with arm-leg BP gradient at baseline or during peak exercise. The TAA/DAO diameter ratio was not associated with higher LV mass/BSA which was independently associated with male gender (p=0.0001) and a larger increase in right arm SBP during peak exercise (p=0.01).

## Conclusions

In a cohort of coarctation patients without significant a residual arm-leg BP gradient, systemic hypertension is common and is associated with hypoplasia of the transverse aortic arch but not with age or type of repair. Patients with TAA hypoplasia also demonstrate a more exaggerated BP response to exercise. Further studies should be undertaken to determine if TAA hypoplasia has a causative role in the development of hypertension or if it is simply a marker for a more diffuse vasculopathy.

## Funding

None.

